# Perspectives on Harmful Algal Blooms (HABs) and the Cyberbiosecurity of Freshwater Systems

**DOI:** 10.3389/fbioe.2019.00128

**Published:** 2019-06-04

**Authors:** David G. Schmale, Andrew P. Ault, Walid Saad, Durelle T. Scott, Judy A. Westrick

**Affiliations:** ^1^School of Plant and Environmental Sciences, Virginia Tech, Blacksburg, VA, United States; ^2^Department of Environmental Health Sciences, University of Michigan, Ann Arbor, MI, United States; ^3^Department of Chemistry, University of Michigan, Ann Arbor, MI, United States; ^4^Bradley Department of Electrical and Computer Engineering, Virginia Tech, Blacksburg, VA, United States; ^5^Department of Biological Systems Engineering, Virginia Tech, Blacksburg, VA, United States; ^6^Lumigen Instrumentation Center, Department of Chemistry, Wayne State University, Detroit, MI, United States

**Keywords:** harmful algal bloom, cyanobacteria, algae, toxin, water security, cybersecurity, drone

## Abstract

Harmful Algal Blooms (HABs) have been observed in all 50 states in the U.S., ranging from large freshwater lakes, such as the Great Lakes, to smaller inland lakes, rivers, and reservoirs, as well as marine coastal areas and estuaries. In 2014, a HAB on Lake Erie containing microcystin (a liver toxin) contaminated the municipal water supply in Toledo, Ohio, providing non-potable water to 400,000 people. Studying HABs is complicated as different cyanobacteria produce a range of toxins that impact human health, such as microcystins, saxitoxin, anatoxin-a, and cylindrospermopsin. HABs may be increasing in prevalence with rising temperatures and higher nutrient runoff. Consequently, new tools and technology are needed to rapidly detect, characterize, and respond to HABs that threaten our water security. A framework is needed to understand cyber threats to new and existing technologies that monitor and forecast our water quality. To properly detect, assess, and mitigate security threats on water infrastructure, it is necessary to envision water security from the perspective of a cyber-physical system (CPS). In doing so, we can evaluate risks and research needs for cyber-attacks on HAB-monitoring networks including data injection attacks, automated system hijacking attacks, node forgery attacks, and attacks on learning algorithms. Herein, we provide perspectives on the research needed to understand both the threats posed by HABs and the coupled cyber threats to water security in the context of HABs.

## Distribution and Transport of HABs in the United States.

**Figure 1:**
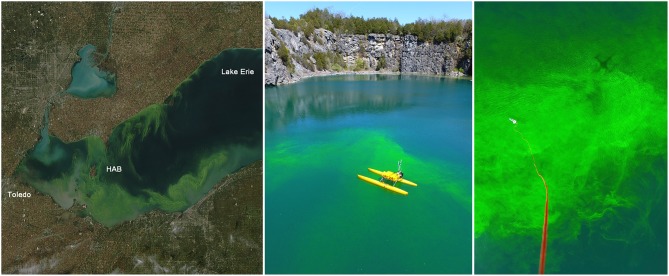
Harmful algal bloom (HAB) in Lake Erie, USA on October 9, 2011 as recorded by Moderate Resolution Imaging Spectroradiometer (MODIS) on the Aqua satellite **(Left)** (courtesy NASA). Technologies with unmanned systems in the water (center panel) and air **(Right)** have the potential to be used to monitor HABs *in situ*. The unmanned systems shown here were tuned to a released fluorescein dye, which has been used a surrogate for HABs (Powers et al., 2018a) (courtesy D. Schmale).

The intensity and frequency of harmful algal blooms (HABs) has increased globally in recent years (Backer et al., 2015). In the U.S., HABs have been observed in variety of freshwater ecosystems including the Great Lakes (Figure 1, left), small inland lakes, and rivers. Consequently, new legislation has been developed to protecting the general public from HABs (National Science Technology Council Subcommittee on Ocean Science Technology, 2016). In 2014, an HAB caused by *Microcystis* at the water treatment plant intake for Toledo, Ohio led to the distribution of non-potable water for multiple days (Steffen et al., 2017). Increasing concerns related to health are not limited to *Microcystis*, as many other genera of cyanobacteria (*Planktothrix, Alexandrium, Anabaena, Cylindrospermopsis, Euglena, etc*.) and associated toxins (anatoxin-a, saxitoxins, cylindrospermopsin, euglenophycin, etc.) have been observed in a range of freshwater systems (Graham et al., 2010; Foss and Aubel, 2015; Loftin et al., 2016; Birbeck et al., 2019). Today, a critical gap still exists on the relationship between human and animal health impact and the range of conditions under which the toxins are produced and the resulting range of toxicities (~50 to >5,000 μg kg^−1^) (Chorus and Bartram, 1999). In 2016, the United States Environmental Protection Agency (EPA) released a draft health advisory for recreational exposure of 4 μg L^−1^ for microcystins and 8 μg L^−1^ for cylindrospermopsin, which are lower than the 20 μg L^−1^ limit recommended by the World Health Organization (WHO) (Environmental Protection Agency, 2016). The lower levels in the draft advisory will result in more frequent exceedance conditions, and highlight that lower concentrations can be harmful. The cyanobacteria and toxins produced by HABs vary considerably within and between freshwater ecosystems, making predictions of HAB formation and toxin production challenging and beyond the capabilities of current models.

Knowledge of the transport of HAB toxins between blooms and the point of human exposure is not well-understood and is crucial for protecting the public. Extracellular material from freshwater cyanobacteria blooms has been observed in the water and in the atmosphere after aerosolization at locations far beyond the edges of HABs (Wood and Dietrich, 2011). For example, extracellular toxins from blooms (e.g., microcystins) have been observed downstream of HABs due to upstream treatment with algicide (Graham et al., 2012). Aerosolized biogenic organicmaterial from freshwater blooms has also been observed in the atmosphere above HABs and transported at least 30 km inland (May et al., 2017, 2018). Thus, it is not only critical to understand when blooms form and produce toxins, but also to understand transport of algal toxins in water and air beyond the bloom.

To improve the security of our freshwater resources it is critical to improve our understanding of HABs and toxin exposures to the point of making predictions that can be used to guide policy and protect public health. This will require transdisciplinary research at the nexus of ecology, atmospheric science, chemistry, and engineering to understand the threats, manage risks, and develop capabilities for reducing exposure to HAB toxins. Challenges to understanding the distribution and transport of HABs include the changing climate, where warmer temperatures facilitate greater HAB formation and changes in nutrient loadings (nitrogen and phosphorus) (Ho and Michalak, 2017; Del Giudice et al., 2018) that are likely to only improve conditions for more intense HABs in many locations across the U.S. (Kosten et al., 2012; Stocker et al., 2013). Remote sensing can provide valuable information regarding the density, extent, and potential impact of blooms that are known to be harmful (Ho and Michalak, 2015; Ho et al., 2017). However, these methods cannot actually determine if an algal bloom is harmful, since they do not monitor the toxins being produced and released. Addressing the challenging of understanding toxin temporal and spatial distributions will require utilizing innovative sampling and direct toxin measurements to improve detection and monitoring capabilities. Such sampling methods must consider the dynamics of the bloom and toxins in relation to the spatial and temporal sampling and testing capacity, to assess the viability of the technology to achieve the goals. For example, unmanned systems (drones) in the air (Benson et al., 2019) and water (Powers et al., 2018a,b) have the potential to be used to detect, track, and sample HABs from the atmosphere and freshwater systems (Figure 1, right). Analytical chemistry approaches can be used to quantify specific HAB toxins from these samples, and to determine chemical signatures to predict toxin production. Ultimately, predictive understanding of freshwater HAB toxin production and transport is essential to improve the security of freshwater systems and protect public health in the many regions with increasingly intense HABs.

## Detection and Characterization of Toxins Associated with HABs

Although there are many classes of cyanotoxins (Janssen, 2019), microcystin (MC), nodularin (NOD), saxitoxin (SXT), anatoxin-a (ANA), and cylindrospermopsin (CYL) are monitored frequently and have been reported in recreational water (Chorus et al., 2000; Graham et al., 2010), drinking water sources (Otten and Paerl, 2015; Steffen et al., 2017), and potable water that was believed to have been treated (Steffen et al., 2017; Davis et al., 2019). Most researchers and water practitioners use commercial semiquantitative cyanotoxin detection technologies because these platforms are inexpensive and need less technical skill (e.g., Humpage et al., 2012; Aranda-Rodriguez et al., 2015). Important limitations of testing technologies within the context of HAB-assessment include throughput capacity, lag times between sampling and results, and the infrastructure/equipment/people needed for implementation.

Commercial semi-quantitative technologies include cyanotoxin class specific enzyme inhibition assays, enzyme-linked immunoassays (ELISAs), and strip tests. Although these technologies are relatively inexpensive, simple and rapid, these assays have a narrow standard dynamic range (0.15 to 5 ppb cyanotoxin) and are not as selective as mass spectrometry (Westrick and Szlag, 2018). Only one enzyme inhibition assay is commercially available, the MC and NOD protein phosphatase inhibition assay (PPIA). Since the MC or NOD Adda amino acid irreversibly binds to the protein phosphatase active site, scientists often refer to the PPIA as a toxicity assay (Carmichael and An, 1999). Limited publications have reported using the commercial protein phosphatase inhibition assay (PPIA); more peer review literature is needed to determine its efficacy (Gaget et al., 2017). Cyanotoxin ELISAs and strip tests are based on polyclonal antibody technology (Humpage et al., 2012; Aranda-Rodriguez et al., 2015). The primary concern with ELISA is that each class of cyanotoxin has several variants or congeners with different toxicities. Usually the antibody is raised against a part of the congener, and does not bind equally to all congeners presenting cross-reactivity effects (Fischer et al., 2001). Antibodies and antibody assays are commercially available for MC, SXT, ANA, and CYL (Westrick and Szlag, 2018). US EPA and several states have required the use of an ELISA where the antibodies are raised against Adda-haptens, providing cross-reactivity to all MC congeners (Fischer et al., 2001). Since all kits use microcystin-specific-LR (MCLR) calibration curves the ELISA units are often called “total” MCLR equivalents (total MCLReq). However, the degree of binding to the antibody does not have a relationship to toxicity (Metcalf et al., 2000), as recent publications suggest that biodegradation and oxidation products interfere with the antibody and provide inaccurate quantitation (Guo et al., 2017; He et al., 2017; Thees et al., 2018).

Liquid chromatography (LC) with various detectors, such as photodiode array (PDA), fluorometer (FL), and tandem mass spectrometer (MS/MS) has been used to quantitate cyanotoxins (Meriluoto et al., 2017). LC/PDA provides detection limits around 0.2 ppb for all MCs because the Adda amino acid produces an ultraviolet spectrum with a maximum absorbance at 238 nm with an extinction coefficient around 39,000 L mol^−1^ cm^−1^ (ISO, 2005). Saxitoxins are derivatized into a fluorescence compound and analyzed by LC/FL. LC/MS/MS methods have been developed for SXT (Onodera et al., 1997), MC (Triantis et al., 2016; Turner et al., 2018), CYL (Guzmán-Guillén et al., 2012), and ANA (Sanchez et al., 2014; Wood et al., 2017). Several MS/MS methods can identify and quantify multiple classes of toxins (Oehrle et al., 2010) with a an expanded dynamic range of about 0.1 to 1,000 ppb beyond the commercial techniques and without the need for solid phase extraction to concentrate the sample. Advancement of a one-step technology, LC/MS/MS with online concentration, enables quantification in under 10 min and provides a dynamic range to 0.005 ppb to 1 ppb (Flores and Caixach, 2017; Birbeck et al., 2019). However, many cyanotoxin quantitation challenges remain. Key issues include known and unknown structural diversity in each cyanotoxin class and between cyanotoxin classes, as well as the lack of reference materials. In order to provide HAB cyber-monitoring, new real-time and passive analytical micro technologies need to be developed and incorporated into unmanned systems for monitoring HABS, such as drones, boats, and buoys (as discussed in the previous section). These unmanned systems can co-locate the sensors with the sampling mechanism, reducing lag times associated with sample transport to a testing facility and improving the applicability of data in near real-time risk assessments.

## Trends in HABs Related to Higher Temperatures and Nutrient Runoff

Balancing food production for the world's growing population while maintaining our water resources is one of society's larger challenges (Foley et al., 2011). Across the globe, our inland freshwater and coastal zones are experiencing widespread eutrophication (Diaz and Rosenberg, 2008), resulting in declining oxygen concentrations (Breitburg et al., 2018), and harmful algal blooms (Anderson et al., 2002). While excess fertilizer use and manure waste has been recognized as an issue for freshwater systems for over two decades (Carpenter et al., 1998), nitrogen and phosphorus runoff into streams and rivers continues to increase. In addition to increasing export, a recent global analysis of riverine nutrient export found a larger proportion of inorganic nitrogen, and phosphorus (Vilmin et al., 2018), altering nutrient ratios relevant for phytoplankton and algal communities.

Nitrogen and phosphorus sources are attributed to fertilizers, animal waste, atmospheric deposition, and municipal sewage. For nitrogen, the Haber-Bosch process has fundamentally altered the nitrogen cycle (Galloway et al., 2004) by providing a mechanism to convert N_2_ to reduced nitrogen for use in fertilizers. Worldwide, fertilizer production continues to increase, largely in the form of urea (Glibert et al., 2006). The primary source of phosphorus fertilizer is from mining. Fossil-fuel combustion and land-conversion over the last century have also provided a source of reactive nitrogen to the atmosphere, which in turn is transported through the atmosphere beyond the emission source leaving no landscape untouched, even the most pristine (Elser et al., 2009).

While fertilizers are required for plant growth, agricultural landscapes are connected to freshwater systems both during dry and wet periods. During storm events and snowmelt, water is transported either across the ground surface or infiltrates through the soil matrix into groundwater and can mobilize nutrients. In regions with poorly draining soils, tile drainage is also used to efficiently remove excess water from a field and can lead to removal of >75% of the water (Van Esbroeck et al., 2016). While these systems maximize food production (Chowdhury et al., 2017), they also short circuit riparian zones and reduce nutrient retention. The dominant forms of nitrogen are generally dissolved, and will move in both surface runoff and into groundwater. Soil erosion is largely thought to be the primary source of phosphorus (Vilmin et al., 2018), although soluble phosphorus is also mobilized and transported through tile drainage (Smith et al., 2015).

Beyond the challenge of excess fertilizer use, the other challenge is our changing climate which may result in higher magnitude storms followed by droughts. While best management practices (BMP) can be adopted, many BMPs may result in nutrient pulses to freshwater systems, which has been shown to increase HAB development (Spatharis et al., 2007). Municipal waste provides a continual nutrient source directly into freshwaters even in times of drought (Mosley, 2015; Vilmin et al., 2018). Thus, while efforts to reduce excess nutrient applications and use of BMPs are needed, our changing climate makes effective solutions challenging (Scavia et al., 2014).

Warmer temperatures are predicted to increase HAB formation and toxin release (O'neil et al., 2012). Two primary mechanisms play a role: increased growth rates and greater stratification and water column stability. Lake experiments have observed higher cyanobacteria growth rates in response to higher temperatures (Liu et al., 2011). In one experimental study, increased water temperature resulted in significant increases in microcystis growth rates (Davis et al., 2019). This research was supported by another study that reported higher growth rates in response to temperature and phosphorus availability (Duan et al., 2009). These HAB responses to warmer water result in greater microcystin toxin releases up to a temperature threshold (Walls et al., 2018). The second mechanism for increased HAB are from enhanced stratification (Joehnk et al., 2008; Rabalais et al., 2009; Paerl et al., 2011). With climate variability, longer, and hotter summers with more frequent droughts are likely to increase water column stability (Mosley, 2015), changing the competition dynamics. Cyanobacteria are able to migrate vertically, providing a competitive advantage (Huisman et al., 2004; Lürling et al., 2013). Future HAB management will require addressing not only nutrient management and runoff, but also thermal regimes and stratification (Paerl et al., 2011), given the body of evidence suggesting the confounding effects from nutrients, temperature, and thermal stratification. Furthermore, integrating monitoring technology that includes the key factors for HAB formation required for forecasting and subsequent HAB and toxin development requires not only improved approaches for toxin quantification and detection but a holistic, secure cyber-physical system (CPS), as described in the next section.

## Frameworks and Research Needs for Water Cyber-Physical Security

HABs pose physical threats on water security. However, to properly detect, assess, and mitigate security threats on water infrastructure, it is imperative to envision water security from the perspective of a cyber-physical system (CPS). Indeed, our national water infrastructure can be seen as a CPS whose physical realm pertains to the physical body of water and the devices that directly interact with it and whose cyber realm pertains to the sensors, smart meters, and other networked apparatus that connect the physical system to the Internet.

**Figure 2:**
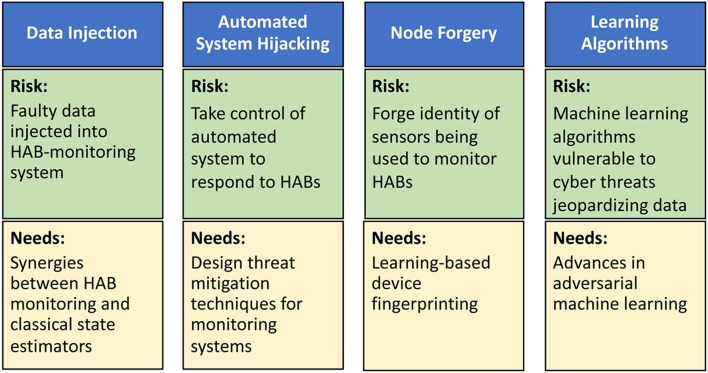
Risks and research needs for cyber-attacks on HAB-monitoring systems including data injection attacks, automated system hijacking attacks, node forgery attacks, and attacks on learning algorithms.

In the context of HABs, we envision four types of cyber-attacks with physical targets: data injection, automated system hijacking, node forgery, and learning algorithms (Figure 2). In data injection attacks, adversaries can inject faulty data, through the HAB monitoring system, to mislead it into underestimating HAB levels. If undetected, such faulty data can potentially lead to a physical catastrophe on the monitored body of water (Moyer et al., 2009). A robust water cybersecurity program is essential to protect public health and prevent service disruptions (Panguluri et al., 2017). Lessons learned from power systems (Sanjab and Saad, 2016) show that such attacks can be done stealthily without being detected by standard state estimators. Meanwhile, automated system hijacking attacks can be launched to take control of any automated system (e.g., an automated drone or sensors) used to respond to rising HAB levels. By taking control of the automated HAB response system, the adversary can derail the system from its original mission thus once again jeopardizing water security. Moreover, the low-cost, small form factor nature of monitoring sensors renders them highly vulnerable to node forgery attacks where an adversary can forge the identity of monitoring sensors and use those captured sensors to jeopardize the integrity of the HAB monitoring data being collected. Finally, the need for data analytics in HAB monitoring will involve machine learning algorithms whose operation will be vulnerable to cyber threats that can jeopardize their input and output data.

Clearly, it is imperative to develop new techniques to mitigate the aforementioned cyber-physical security threats (Figure 2). To this end, as shown in Sanjab and Saad (2016) and Ferdowsi et al. (2017), one can leverage tools from game theory to understand how defenders and attackers interact over a water CPS and, therefore, identify potential vulnerabilities and optimal cyber-physical defense strategies. This theoretical analysis can then be used to develop various software/hardware solutions, such as improved estimation algorithms and robust control mechanisms (Ferdowsi et al., 2018), to mitigate data injection and autonomous system hijack threats. To deal with node forgery attacks, the use of learning-based device fingerprinting (Ferdowsi and Saad, 2018) can be particularly apropos. In addition, advances in adversarial machine learning (McDaniel et al., 2016) can provide tools to develop robust data analytics in water systems. However, additional research is needed to tailor existing solutions to the unique properties of water systems.

In terms of data injection attacks, research is needed to understand the synergies and distinctions between HAB monitoring systems and classical state estimators, such as those used in power systems. This understanding is necessary to devise HAB-specific solutions for mitigating data injection attacks. Water infrastructure will not only depend on the cyber infrastructure, but it will also be interconnected with other infrastructure in a city. This interdependence and its impact on CPS security must be identified and analyzed. In terms of automated system hijacking attacks, it is necessary to first introduce HAB-centric automated systems that can effectively help in monitoring and treating HAB-affected bodies of waters. Once such systems are in place, one can better design threat mitigation techniques that are tailored to those systems by leveraging on lessons learned from other fields, such as autonomous vehicles (Ferdowsi et al., 2018).

In summary, new analytical tools that can build on existing techniques such as graph theory and game theory, are needed to devise realistic models for water systems, in general, and HAB-centric water monitoring systems in particular. Such models must capture the physical dynamics of the system as well as the cyber-interconnections. By devising such models, potential vulnerabilities can be better identified and new strategies for securing the system can be devised. Research developing a convergent paradigm at the nexus of engineering, ecology, and chemistry is needed to understand threats, manage risks, and develop capabilities for water cyberbiosecurity.

## Author Contributions

All authors listed have made a substantial, direct and intellectual contribution to the work, and approved it for publication.

### Conflict of Interest Statement

The authors declare that the research was conducted in the absence of any commercial or financial relationships that could be construed as a potential conflict of interest.
